# Oral Administration of Vitamin D3 Prevents Corneal Damage in a Knock-Out Mouse Model of Sjögren’s Syndrome

**DOI:** 10.3390/biomedicines11020616

**Published:** 2023-02-18

**Authors:** Maria Consiglia Trotta, Hildegard Herman, Cornel Balta, Marcel Rosu, Alina Ciceu, Bianca Mladin, Carlo Gesualdo, Caterina Claudia Lepre, Marina Russo, Francesco Petrillo, Gorizio Pieretti, Francesca Simonelli, Settimio Rossi, Michele D’Amico, Anca Hermenean

**Affiliations:** 1 Department of Experimental Medicine, University of Campania “Luigi Vanvitelli”, Via Santa Maria di Costantinopoli 16, 80138 Naples, Italy; 2 “Aurel Ardelean” Institute of Life Sciences, Vasile Goldis Western University of Arad, 86 Revolutiei Av., 310414 Arad, Romania; 3 Multidisciplinary Department of Medical, Surgical and Dental Sciences, University of Campania “Luigi Vanvitelli”, Via Luigi de Crecchio 6, 80138 Naples, Italy; 4 PhD Course in Translational Medicine, Department of Experimental Medicine, University of Campania “Luigi Vanvitelli”, 80138 Naples, Italy

**Keywords:** vitamin D, Sjögren’s syndrome, cornea, dry eye, thrombospondin-1 knock-out mice, tumor necrosis factor alpha converting enzyme

## Abstract

Background: Vitamin D deficiency has been associated with dry eye development during Sjögren’s syndrome (SS). Here, we investigated whether repeated oral vitamin D3 supplementation could prevent the corneal epithelium damage in an SS mouse model. Methods: 30 female mouse knock-out for the thrombospondin 1 gene were randomized (six per group) in untreated mice euthanized at 6 weeks as negative control (C−) or at 12 weeks as the positive control for dry eye (C+). Other mice were sacrificed after 6 weeks of oral vitamin D3 supplementation in the drinking water (1000, 8000, and 20,000 IU/kg/week, respectively). Results: The C+ mice showed alterations in their corneal epithelial morphologies and thicknesses (*p* < 0.01 vs. C−), while the mice receiving 8000 (M) and 20,000 (H) IU/kg/week of vitamin D3 showed preservation of the corneal epithelium morphology and thickness (*p* < 0.01 vs. C+). Moreover, while the C+ mice exhibited high levels and activity of corneal tumor necrosis factor alpha converting enzyme (TACE), neovascularization and fibrosis markers; these were all reduced in the M and H mice. Conclusions: Oral vitamin D3 supplementation appeared to counteract the negative effect of TACE on corneal epithelium in a mouse model of SS-associated dry eye.

## 1. Introduction

Sjögren’s syndrome (SS) is a multiorgan disorder that is more common in females than in males, with a female to male ratio of ~9:1 [1,2,3,4,5].

The cornea is one of the targets undergoing SS ocular damaging consequences. This refers to damage of the Meibomian glands, corneal melt/perforation, uveitis, scleritis, retinal vasculitis, and optic neuritis [6], together with disease activity in cutaneous, respiratory, renal, articular, muscular, peripheral nervous system, central nervous system, hematologic, glandular, constitutional, lymphadenopathic, and biological organs [7]. Several mediators have been identified as being responsible for the damaging effects, primarily those residing in the oxidative-inflammatory response [8]. Among these, tumor necrosis factor alpha (TNF-α) has been considered a key player in the development of cell, tissue, and organ damage for several years, and therefore, it appeared to be an important target for ocular therapies. However, side effects have emerged from clinical trials testing anti-TNF-α and anti-interleukin 1 beta (Il-1β) drugs [9]. In contrast, the prevention of the TNF-α transmembrane cleavage by the inhibition of TACE (the TNF-α-converting enzyme) could be a valid alternative [9] for reducing the incidence of the cytokines on the inflammatory burden, which causes organ damage.

In humans, one of the factors that worsens SS symptoms and corneal damage is vitamin D deficiency [10,11]. This deficiency is responsible for shorter tear breakup times, lower Schirmer’s test scores for SS individuals, and higher ocular surface disease index scores [11]. Vitamin D deficiency affects corneal epithelium and endothelium [12] by producing local inflammation, altering the ocular surface, and causing dry eye [13]. Conversely, vitamin D may relieve such symptoms by inhibiting the key mediators of localized inflammation and stimulating the release of antioxidant cytokines in the tears and cornea [13,14]. These actions can be exerted through a reduction in corneal TACE activity or levels, since vitamin D has been shown to inhibit TACE in other experimental settings [15,16,17].

Therefore, the present study investigated whether the repeated oral administration of vitamin D3 could prevent SS corneal damage in an SS mouse model (thrombospondin-1 knock-out (TSP-1 KO) mice). A possible reduction in corneal TNF-α transmembrane cleavage, as monitored by corneal TACE levels and activity, was investigated after vitamin D3 pretreatment, and the effects of vitamin D3 supplementation on corneal epithelium morphology, integrity, and neovascularization [18,19] were evaluated by analyzing the corneal levels of vascular endothelial growth factor (VEGFA), vascular endothelial growth factor receptor 2 (VEGFR2), transforming growth factor alpha (TGF-α), and transforming growth factor beta (TGF-β). All of these mediators are considered important markers of SS and are induced by TACE [20,21].

## 2. Materials and Methods

### 2.1. Animal Design

TSP-1 female mice (006141-F, B6.129S2-Thbs1<tm1Hyn>/J HOM Homozygous for Thbs1<tm1Hyn>; Bio Zyme SRL, Cluj-Napoca, Romania), knock-outs for thrombospondin 1 gene (Thbs1), were used as the animal model for SS-associated dry eye [22,23,24]. Due to the protective role of TSP-1 in ocular inflammatory processes [25,26], the mice had been shown to develop SS-associated ocular complications at 12 weeks from birth [23,24]. The TSP-1 female mice were randomized into 5 experimental groups (N = 6 mice per group): (I) TSP-1 KO mice euthanized at 6 weeks of age as a negative control for SS-associated dry eye (C−); (II) TSP-1 KO mice euthanized at 12 weeks of age as a positive control for SS-associated dry eye (C+); and (III-IV-V) TSP-1 KO mice supplied with a low (L), medium (M), and high (H) dose of vitamin D3 (1000, 8000, and 20,000 IU/kg of body weight/week, respectively) starting from 6 weeks of age. These doses were chosen to obtain a dose-response in our setting, in line with previous experiments in mice [27,28,29]. Vitamin D3 (cholecalciferol, C9756-5G—Sigma, Milano, Italy) was dissolved in ethanol and then added to drinking water [30,31,32] for 6 weeks, and then, the mice were sacrificed at week 12 under ketamine and xylazine anesthesia. Serum samples were obtained from 0.2 to 0.25 mL of blood samples taken from the tail vein, collected into a heparinized Eppendorf vial, left for 30 min at room temperature, and then centrifuged at 3000× *g* for 10 min at 4 °C to collect the supernatants [33]. The study subjects’ eyes were enucleated to isolate the corneas from the other ocular tissues. After the corneas were dissected in cooled phosphate buffer saline (PBS), 7 corneas were suddenly immersed in a suitable buffer for the histological evaluations, while 5 corneas were immediately frozen in liquid nitrogen and stored at −80 °C for subsequent biochemical analysis [34].

All animal procedures were approved by the Animal Ethics Committee of the Vasile Goldiș Western University of Arad (11/18.02.2022) and the Authorization of the National Sanitary Veterinary and Food Safety Authority Romania (ANSVSA) (1583/28.03.2022).

### 2.2. Vitamin D Levels

The levels of 1,25-Dihydroxyvitamin D3 (DHVD3), the active form of vitamin D3 [35], were evaluated in the TSP-1 KO mice sera to monitor the different serum DHVD3 levels as an example of the different vitamin D3 doses reaching the plasma. The DHVD3 serum levels were also assessed in female Balb-c mice (6 and 12 weeks of age) as healthy controls (Table S1). A commercially available enzyme-linked immunosorbent assay (ELISA) (MBS2602146, MyBiosource, San Diego, CA, USA) was used, following the manufacturer’s protocol for serum sample preparation.

### 2.3. Histology

Eye samples were fixed in a 4% formaldehyde solution in PBS, embedded in paraffin, and then stained using Gomori’s trichrome (GT) stain kit (38016SS1, Leica, Allendale, NJ, USA), according to the instructions given by the Bio-Optica staining kit (Italy). An Olympus BX43 microscope with a digital camera (Olympus XC30, Hamburg, Germany) was used for examining the sections [36]. The central corneal thickness (µm) was determined by measuring the distance between 2 peaks, representing the corneal epithelium and endothelium, using CellSens Dimension Imaging Software (v 1.10, Olympus, Hamburg, Germany). The measurements were performed in triplicate for each eye. The values were averaged and reported as the means and standard deviations.

### 2.4. Immunohistochemistry

We used 5 µm eye sections for the immunohistochemistry after paraffin imbibition, deparaffinization, and rehydration. The primary antibodies used were rabbit polyclonal TGF-β1 (sc-146; Santa Cruz Biotechnology; Dallas, TX, USA) and Smad2/3 (sc-133098; Santa Cruz Biotechnology; Dallas, TX, USA). These were diluted to 1:200 and incubated overnight at 4 °C. Immunoreactions were detected using a Novocastra Peroxidase/DAB kit (Leica Biosystems, Nussloch, Germany). The negative control sections were stained with irrelevant immunoglobulins and analyzed under a bright-field microscope [37]. The percentage of positive-stained area/total area was quantified using ImageJ software 1.47.

### 2.5. Immunofluorescence

For the immunofluorescence, eye sections were deparaffinized and rehydrated before being incubated overnight in Epitope Retrieval Solution (Leica Biosystems Inc., Buffalo Grove, IL, USA) at 60 °C. Then, the sections were blocked at room temperature for 40 min with a solution containing PBS, 2% bovine serum albumin (BSA) (ABIN934476, Antibodies-online), and 0.1% Triton-X100 (X100, Sigma-Aldrich, St. Louis, MO, USA). Primary rabbit anti-VEGFR2 (bs-10412R, 1:100 dilution; Bioss, Woburn, MA, USA) and rabbit anti-TACE (bs-4236R; 1:100 dilution; Bioss, USA) antibodies were diluted in the primary antibody diluting buffer (Bio-Optica, Milano, Italy) and incubated for 2 h at room temperature. Slides were incubated for 30 min at room temperature in the dark with Cy5-labeled goat anti-rabbit IgG secondary antibody (A10523; Thermo Fisher Scientific Inc., Rockford, IL, USA) and diluted 1:500 in PBS before being counterstained with 4′,6-diamidino-2-phenylindole (DAPI) for 5 min. CC/Mount aqueous mounting medium (Sigma-Aldrich, St. Louis, MO, USA) was used to mount the slides. Images were acquired using a Leica TCS SP8 laser scanning confocal microscope. The percentage of positive-stained area/total area was quantified using ImageJ software.

### 2.6. Western Blotting

The protein content was obtained by homogenizing the corneas in RIPA lysis buffer (R0278, Merck, Bari, Italy) containing protease and phosphatase inhibitors (PPC1010, Merck, Bari, Italy) and then centrifuging the samples for 10 min at 13,000× *g* at 4 °C. The protein concentration in the supernatants was assessed using a Bio-Rad Protein Assay (500-0006, Bio-Rad Laboratories, Segrate, Italy) [34]. From each cornea, more than 8 µg of total proteins were extracted from each cornea, in line with previous evidence [38].

The Western blotting assay was performed on 4.0 μg of extracted proteins, which were separated using sodium dodecyl sulphate-polyacrylamide gel electrophoresis (SDS-PAGE) (10% polyacrylamide) before being electro-transferred to the polyvinylidene difluoride membranes. Then, the membranes were blocked at room temperature for 1 h with the blocking solution composed of tris-buffered saline (TBS, 1X—12498S, Euroclone, Milan, Italy), Tween 20 (0.01%—P1379; Sigma-Aldrich, Milan, Italy), and non-fat dry milk (5%—EMR180500; Euroclone SpA, Milan, Italy). The following primary antibodies, dissolved in blocking solution (3% non-fat dry milk), were used for the overnight incubation at 4 °C: anti-VEGFA (1:500, sc-507—Santa Cruz, CA, USA) and -Glyceraldehyde-3-Phosphate Dehydrogenase (GAPDH) (C-2) (1:200, sc-8432, Santa Cruz Biotech, Santa Cruz, CA, USA). Anti-mouse (1:2000, sc-2005; Santa Cruz Biotech, CA, USA) and anti-rabbit (1:2000, sc-2004; Santa Cruz Biotech, CA, USA) secondary antibodies, conjugated with horseradish peroxidase and diluted in blocking solution (3% non-fat dry milk), were incubated at room temperature for 1 h. The immunoreactive signals detected were visualized with an ECL system (35055, Thermo Fisher Scientific, Rodano, Italy). Then, ChemiDoc-It 5000 and VisionWorks Life Science Image Acquisition and Analysis software 81-0254-01 (UVP, Upland, CA, USA) were used to quantify the immunoreactive signals and to normalize them with the GAPDH protein levels. The results were expressed as densitometric units (DU). Uncropped images of the representative VEGFA and GAPDH Western blotting membranes are shown in Figure S1.

### 2.7. ELISAs

The levels of TNF-α as a marker of TACE activity [38], TGF-α as a marker of corneal epithelial derangement [39,40], and TGF-β as marker of corneal fibrosis [41] were detected in the mice corneas using ELISA assays (Mouse TNF-alpha EM0183 and Mouse TGF-alpha EM1405, Fine Test, China; Mouse TGF-β MBS160136, MyBiosource, CA, USA), according to the manufacturer’s protocols.

### 2.8. Statistical Analysis

The results are reported as the means ± standard errors (SD). Statistical significance was assessed with one-way analysis of variance (ANOVA), followed by Tukey’s multiple comparisons test, using GraphPad Prism (6.0 GraphPad Software, La Jolla, CA, USA). *p*-values of <0.05 were considered statistically significant.

## 3. Results

### 3.1. Serum Levels of DHVD3 in the TSP-1 KO Mice

**Figure 1:**
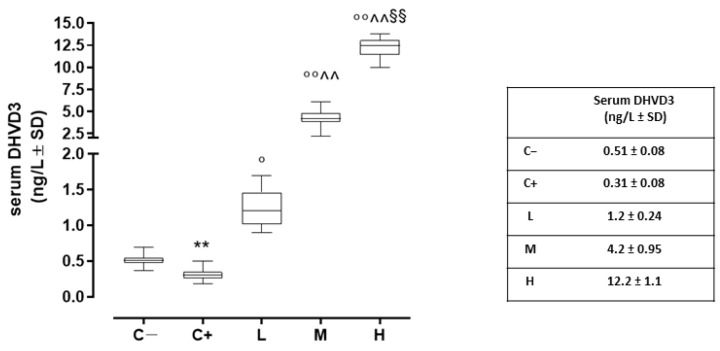
Serum DHVD3 levels (ng/L) in the TSP-1 KO mice euthanized at 6 weeks of age as a negative control for SS-associated dry eye (C; TSP-1 KO mice euthanized at 12 weeks of age as a positive control for SS-associated dry eye (C+); and TSP-1 KO mice supplied with a low (L), medium (M), and high (H) dose of vitamin D3 (1000, 8000, and 20,000 IU/kg/week, respectively) from week 6 to week 12 of age. N = 6 mice per group. ** *p* < 0.01 vs. C−; ° *p* < 0.05 and °° *p* < 0.01 vs. C+; ^^ *p* < 0.01 vs. L; ^§§^ *p* < 0.01 vs. M.

Compared to the negative control (C−; 0.51 ± 0.08 ng/L), the serum DHVD3 (the active metabolite of vitamin D3) was found to be significantly downregulated in the untreated TSP-1 KO mice that developed dry eye (C+) (0.31 ± 0.08 ng/L, *p* < 0.01 vs. C−). After 6 weeks, the serum DHVD3 was significantly increased in the TSP-1 KO mice pretreated with 1000 IU/kg/week (L; 1.2 ± 0.24 ng/L, *p* < 0.05 vs. C+), 8000 IU/kg/week (M; 4.2 ± 1.0 ng/L, *p* < 0.01 vs. C+), and 20,000 IU/kg/week (H; 12.2 ± 1.2 ng/L, *p* < 0.01 vs. C+) (Figure 1).

### 3.2. Effect of Vitamin D3 Pretreatment on Corneal Epithelium in the TSP-1 KO Mice

**Figure 2:**
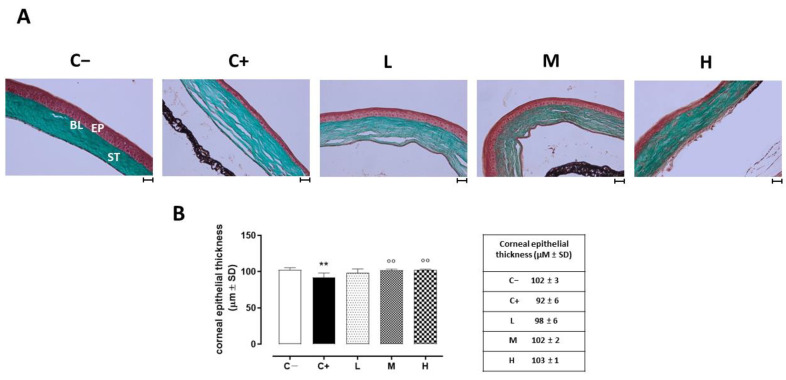
(**A**) Corneal GT staining in the TSP-1 KO mice euthanized at 6 weeks of age as a negative control for SS-associated dry eye (C−); TSP-1 KO mice euthanized at 12 weeks of age as a positive control for SS-associated dry eye (C+); and TSP-1 KO mice supplied with low (L), medium (M), and high (H) doses of vitamin D3 (1000, 8000, and 20,000 IU/kg/week, respectively) from week 6 to week 12 of age. EP: corneal epithelium; BL: Bowman’s layer; ST: stroma. Scale bar: 20 µm; magnification 40×. (**B**) Central corneal epithelial thickness (µm ± SD) in the same experimental groups. N = 7 corneas per group. ** *p* < 0.01 vs. C−; °° *p* < 0.01 vs. C+.

The histopathology of the corneas before and after treatment was examined under optic microscopy. Representative images of the corneal sections in (GT) staining are shown in Figure 2A. The control corneas showed normal morphologies. Morphological changes were noted in the epithelial layers of the dry eye-affected corneas (C+), such as basal epithelial layers with enlarged cells, irregular shapes, and hyperchromatic nuclei. The central corneal thickness decreased (92 ± 6 µm, *p* < 0.01 vs. C−) compared to the C− group (102 ± 3 µm). Under the vitamin D supplementation, the epithelial thickness and the corneal structure were restored in a dose-dependent manner, being similar to the control (normal) corneas for the higher dose (M: 102 ± 2 µm, *p* < 0.01 vs. C+; H: 103 ± 1 µm, *p* < 0.01 vs. C+). Conversely, the lower dose of vitamin D supplementation was not effective in restoring the corneal epithelial thickness (L: 98 ± 6 µm, *p* > 0.05 vs. C+) (Figure 2B).

### 3.3. Effect of Vitamin D3 Pretreatment on Corneal TACE Expression and Activity

**Figure 3:**
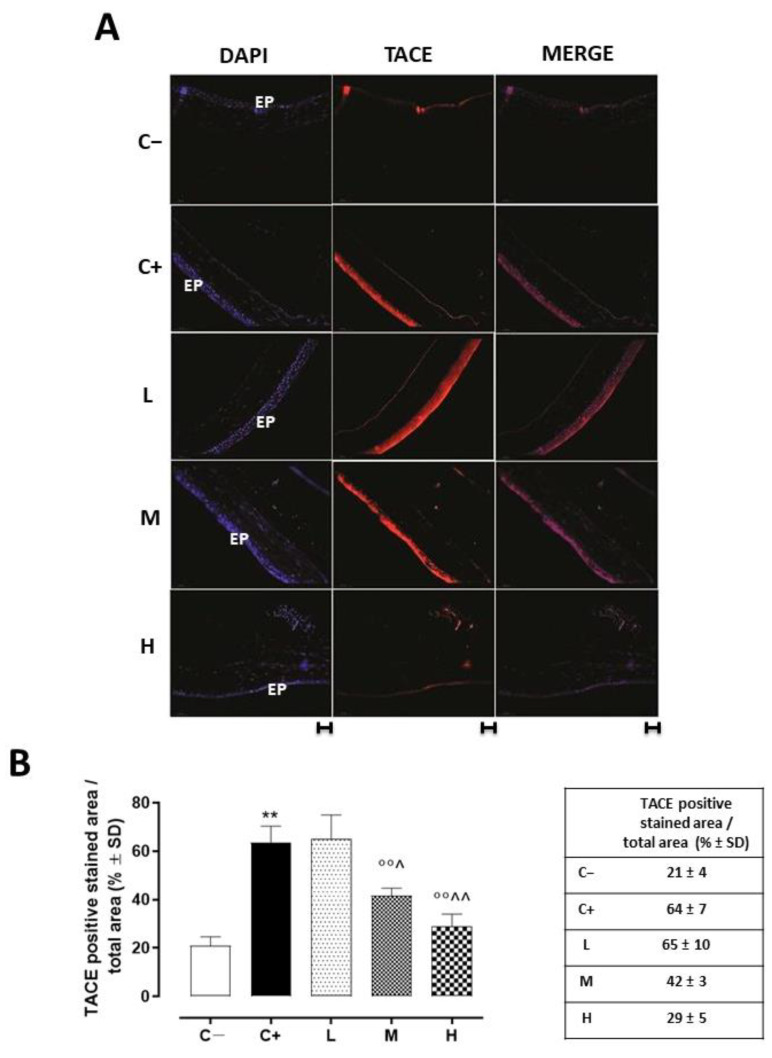
(**A**) Representative immunofluorescence images of TACE staining (red) and (**B**) relative quantization, expressed as percentages of TACE positive cells (red)/total cells counted (blue) in the TSP-1 KO mice euthanized at 6 weeks of age as a negative control for SS-associated dry eye (C−); TSP-1 KO mice euthanized at 12 weeks of age as a positive control for SS-associated dry eye (C+); and TSP-1 KO mice supplied with low (L), medium (M), and high (H) doses of vitamin D3 (1000, 8000, and 20,000 IU/kg/week, respectively) from week 6 to week 12 of age. N = 7 corneas per group; EP: corneal epithelium; scale bar 20 µm; magnification 40×. ** *p* < 0.01 vs. C−; °° *p* < 0.01 vs. C+; ^ *p* < 0.05 and ^^ *p* < 0.01 vs. L.

Corneal TACE staining was evident in the corneal epithelium layer. This was significantly increased in the C+ group (64 ± 7%, *p* < 0.01 vs. C−) and in the L mice (65 ± 10%, *p* > 0.05 vs. C+) (Figure 3A,B).

**Figure 4:**
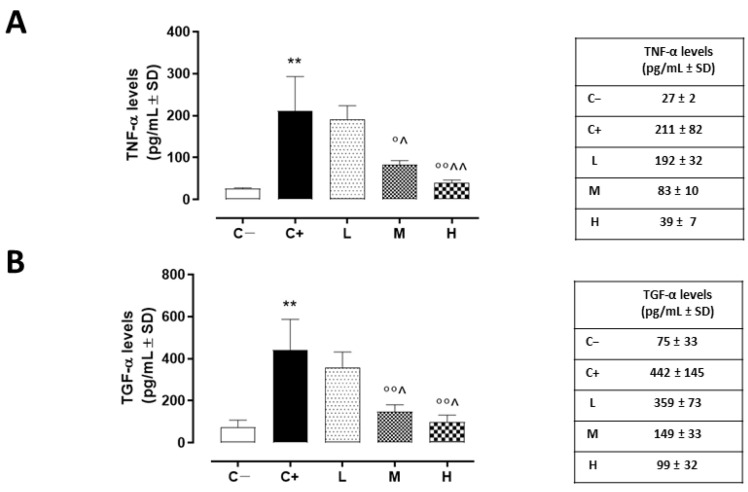
(**A**) Corneal TNF-α (pg/mL ± SD) and (**B**) TGF-α (pg/mL ± SD) levels in the TSP-1 KO mice euthanized at 6 weeks of age as a negative control for SS-associated dry eye (C−); TSP-1 KO mice euthanized at 12 weeks of age as a positive control for SS-associated dry eye (C+); and TSP-1 KO mice supplied with low (L), medium (M), and high (H) doses of vitamin D3 (1000, 8000, and 20,000 IU/kg/week, respectively), from week 6 to week 12 of age. N = 5 corneas per group. ** *p* < 0.01 vs. C−; ° *p* < 0.05 and °° *p* < 0.01 vs. C+; ^ *p* < 0.05 and ^^ *p* < 0.01 vs. L.

The same trend was detected for corneal TACE activity, measured as TNF-α levels, which were higher in the corneas of the C+ group (211 ± 81 pg/mL, *p* < 0.01 vs. C−) and in the L mice (192 ± 32 pg/mL, *p* > 0.05 vs. C+) (Figure 4A).

A significant reduction was evident in the corneal TACE levels of the M and H mice (42 ± 3% and 29 ± 5%, respectively, with both *p*-values being <0.01 vs. C+) (Figure 3B), as well as in the corneal TNF-α levels of the same groups (83 ± 10 pg/mL, *p* < 0.05 vs. C+ and 39 ± 7 pg/mL, *p* < 0.01 vs. C+) (Figure 4A).

### 3.4. Effect of Vitamin D3 Pretreatment on Corneal Epithelium Derangement, Marked by TGF-α

TGF-α, another target of TACE activity and a marker of corneal epithelium derangement, was found to be significantly increased in the C+ mice (442 ± 145 pg/mL, *p* < 0.01 vs. C−). In addition, the L mice exhibited elevated corneal TGF-α levels (359 ± 73 pg/mL, *p* > 0.05 vs. C+). Conversely, there was a decrease in these same levels in the TSP-1 KO mice receiving medium and high doses of vitamin D3 pretreatment (149 ± 33 and 99 ± 33 pg/mL, respectively, with both *p*-values being <0.01 vs. C+) (Figure 4B).

### 3.5. Effect of Vitamin D3 Pretreatment on the Neovascularization of the Corneal Epithelium

**Figure 5:**
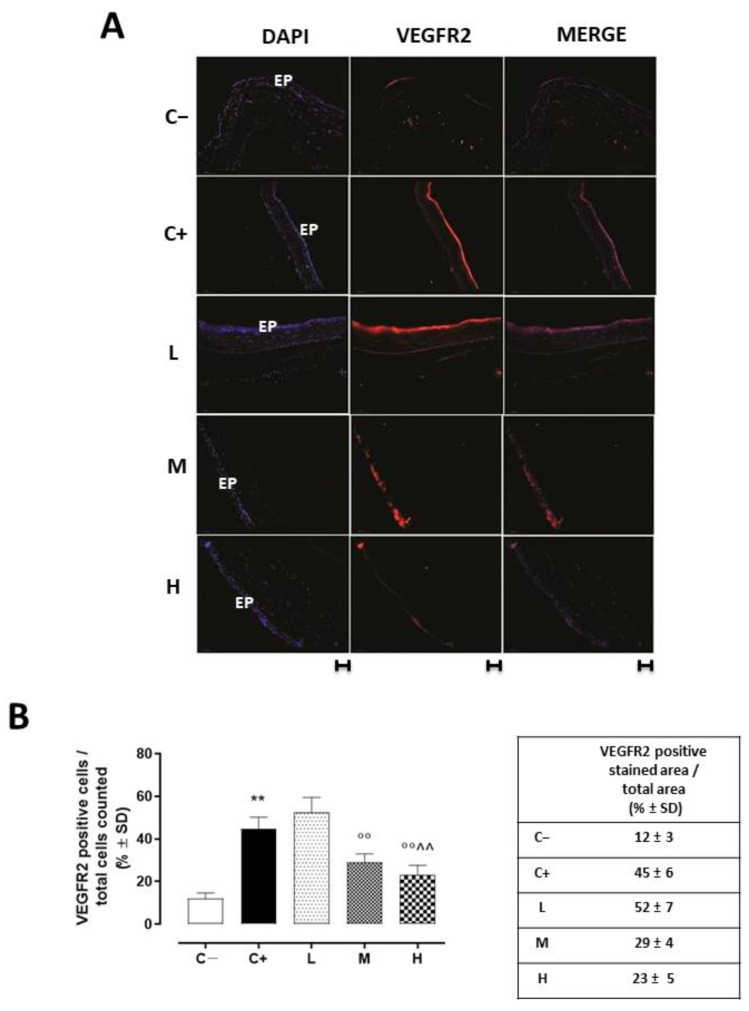
(**A**) Representative immunofluorescence images of the VEGFR2 staining (red) and (**B**) relative quantization, expressed as percentage of the VEGFR2 positive stained area (red)/total cells counted (blue) in the TSP-1 KO mice euthanized at 6 weeks of age as a negative control for SS-associated dry eye (C−); TSP-1 KO mice euthanized at 12 weeks of age as a positive control for SS-associated dry eye (C+); and TSP-1 KO mice supplied with low (L), medium (M), and high (H) doses of vitamin D3 (1000, 8000, and 20,000 IU/kg/week, respectively) from week 6 to week 12 of age. N = 7 corneas per group; EP: corneal epithelium. Scale bar: 20 µm; magnification 40×. ** *p* < 0.01 vs. C−; °° *p* < 0.01 vs. C+; ^^ *p* < 0.01 vs. L.

VEGFR2 staining (red) was evident in the corneal epithelia of the untreated TSP-1 KO mice that developed dry eye compared to the C− group (45 ± 5%, *p* < 0.01 vs. C−), and this staining was not altered in the L mice (52 ± 7%, *p* > 0.05 vs. C+) (Figure 5A,B).

**Figure 6:**
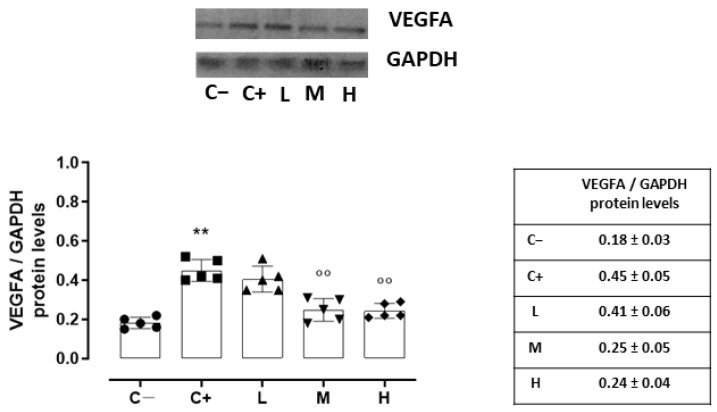
Representative Western blot images for corneal VEGFA and GAPDH, with relative quantization (both VEGFA and GAPDH values were detected as densitometric units ± SD) in the TSP-1 KO mice euthanized at 6 weeks of age as a negative control for SS-associated dry eye (C−); TSP-1 KO mice euthanized at 12 weeks of age as a positive control for SS-associated dry eye (C+); and TSP-1 KO mice supplied with low (L), medium (M), and high (H) doses of vitamin D3 (1000, 8000, and 20,000 IU/kg/week, respectively) from week 6 to week 12 of age. N = 7 corneas per group; EP: corneal epithelium. N = 5 corneas per group. ** *p* < 0.01 vs. C−; °° *p* < 0.01 vs. C+.

Accordingly, the VEGFA protein levels, quantized on the glyceraldehyde-3-phosphate dehydrogenase (GAPDH) protein levels, were increased in the C+ (0.45 ± 0.05, *p* < 0.01 vs. C−) and L (0.41 ± 0.06, *p* > 0.05 vs. C+) groups (Figure 6).

A significant reduction was evident in the corneal VEGFR2 and VEGFA levels of the TSP-1 KO mice receiving medium (29 ± 4% and 0.25 ± 0.05, respectively, with both *p*-values being <0.01 vs. C+) and high doses (23 ± 5% and 0.24 ± 0.04, respectively, with both *p*-values being <0.01 vs. C+) of the vitamin D3 pretreatments (Figure 5 and Figure 6).

### 3.6. Effect of Vitamin D3 Pretreatment on Corneal Epithelium Fibrosis and Mesenchymal Transition

**Figure 7:**
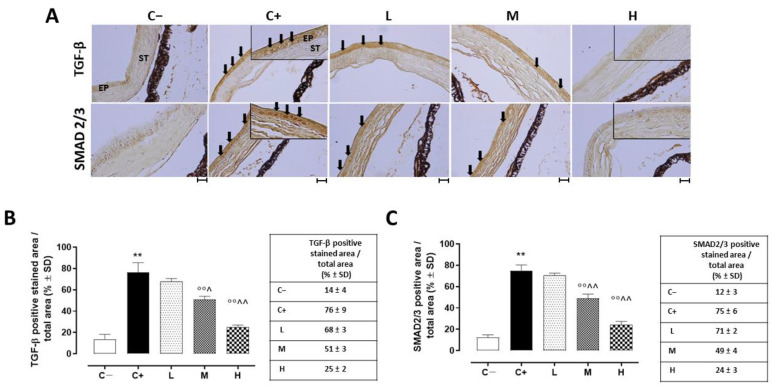
(**A**) Representative immunohistochemistry images of corneal TGF-β and SMAD2/3 and the relative quantization of TGF-β (**B**) and SMAD2/3 (**C**) staining (black arrows), expressed as percentages ± SDs of the positive stained area/total area in the TSP-1 KO mice euthanized at 6 weeks of age as a negative control for SS-associated dry eye (C−); TSP-1 KO mice euthanized at 12 weeks of age as a positive control for SS-associated dry eye (C+); and TSP-1 KO mice supplied with low (L), medium (M), and high (H) doses of vitamin D3 (1000, 8000, and 20,000 IU/kg/week, respectively) from week 6 to week 12 of age. N = 7 corneas per group; EP: corneal epithelium; ST: stroma. Scale bar 20 µm; magnification 40×. ** *p* < 0.01 vs. C−; °° *p* < 0.01 vs. C+; ^ *p* < 0.05 and ^^ *p* < 0.01 vs. L.

A strong TGF-β immunoreactivity (black arrows) was evident in the corneal epithelia of the C+ mice (76 ± 9%, *p* < 0.01 vs. C−) in comparison to the C− group (Figure 7A,B).

**Figure 8:**
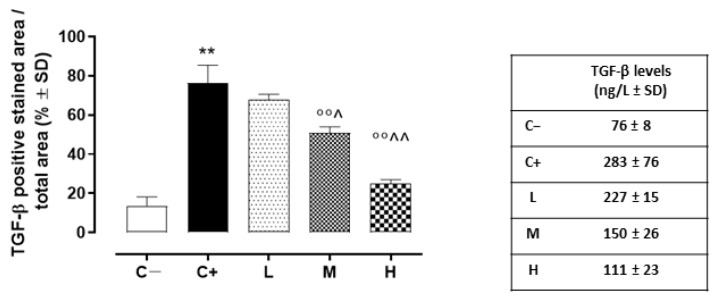
Corneal TGF-β levels (ng/L ± SD) in the TSP-1 KO mice euthanized at 6 weeks of age as a negative control for SS-associated dry eye (C−); TSP-1 KO mice euthanized at 12 weeks of age as a positive control for SS-associated dry eye (C+); and TSP-1 KO mice supplied with low (L), medium (M), and high (H) doses of vitamin D3 (1000, 8000, and 20,000 IU/kg/week, respectively) from week 6 to week 12 of age. N = 5 corneas per group. ** *p* < 0.01 vs. C−; °° *p* < 0.01 vs. C+; ^ *p* < 0.05 and ^^ *p* < 0.01 vs. L.

In addition, the TGF-β levels detected in the C+ group’s corneas (273 ± 76 ng/L; *p* < 0.01 vs. C+) were elevated compared to those of the C- mice (Figure 8).

While the L mice showed no differences in corneal TGF-β staining (68 ± 3%, *p* > 0.05 vs. C+) and levels (228 ± 16 ng/L, *p* > 0.05 vs. C+) compared to the C+ group, both the TGF- β immunoreactivity and levels were found to be significantly reduced in the M (51 ± 3% and 150 ± 27 ng/L, respectively, with both *p*-values being <0.01 vs. C+) and H groups (25 ± 2% and 111 ± 23 ng/L, respectively, with both *p*-values being <0.01 vs. C+) (Figure 7A,B and Figure 8). Accordingly, the staining of SMAD2/3, involved in TGF-β signaling, was marked in the corneal epithelia of the C+ (75 ± 6%, *p* < 0.01 vs. C−) and L mice (71 ± 2%, *p* > 0.05 vs. C+), while it was decreased in the H and M mice (49 ± 4% and 24 ± 3%, respectively, with both *p*-values being <0.01 vs. C+) (Figure 7A,C).

## 4. Discussion

It was demonstrated here that the TSP-1 KO mice, who were genetically predisposed to develop SS ocular symptoms and morphological alterations after 12 weeks of age [42,43], were characterized by a damaged corneal epithelial layer and showed an abnormal neovascularization. This was paralleled by high TACE levels and activity.

TACE is a constitutive multi-domain type I transmembrane protein belonging to the A Disintegrin And Metalloprotease (ADAM) family [42,44]. It is strictly involved in corneal epithelium damage and detachment [43,45]. When activated, TACE generates the soluble forms of TNF-α, TGF-α, and other proteins from their membrane-bound precursors (a phenomenon called shedding) through intracellular kinase activities. These mediators have fundamental roles in corneal epithelium inflammation and derangement [39,40,46]. Therefore, TACE inhibition has been explored as a therapeutic tool for preserving corneal structures and functions [43,45].

Interestingly, the TACE levels and activity seem to be inhibited in renal osteodystrophy, testicular torsion, and secondary hyperparathyroidism by vitamin D [15,16,17], which deficiency has been considered as a risk factor for the development of SS-ocular complications [10,11,12,13,14,47]. Of interest, the treatment of human corneal epithelial cells with vitamin D lead to an improved antibacterial response in a model of ocular surface disease [48,49] and to a reduced inflammatory state in a model of dry eye disease [50,51].

The positive effects of vitamin D3 on corneal derangement induced by SS were observed also in our experimental setting, showing an improved corneal epithelial morphology in TSP1-KO mice supplemented with oral vitamin D3. This was in line with a previous study analyzing the actions of vitamin D3 on corneal epithelium in a rat model of dry eye [52].

Moreover, in accordance with the results showing TACE inhibition by vitamin D, here we report for the first time that the high corneal TACE levels and activity showed by the TSP-1 KO mice were prevented by repeated oral vitamin D3 supplementation for 6 weeks. This was obtained with high vitamin D3 doses, which were previously reported to be efficient for increasing mouse bone mass and strength [27], attenuating frailty progression during aging [53], and preventing hypocalcemia and osteomalacia [28].

We also analyzed, for the first time, the effect of orally taken vitamin D3 on the corneal levels of TGF-α and TGF-β. TGF-α is a target of TACE that has a detrimental effect on corneal epithelial degeneration when upregulated [40,54]. Similarly, TGF-β is a marker of corneal fibrosis and epithelial–mesenchymal transition [41,55], both of which seem to be induced by TNF-α increases [56]. In both cases, high doses of vitamin D3 supplementation diminished the abnormally increased presence of the growth factors, which was in line with previous evidence from different experimental settings [52,57,58,59].

On another note, oral vitamin D3 also decreased the corneal levels of the proteins VEGFA and the receptor VEGFR2, which were highly expressed in the corneas of the TSP1-KO mice. The reduction of both VEGFR2 and VEGFA in the cornea of animals pretreated with vitamin D3 was in line with a previous study reporting a moderate inhibition of corneal neovascularization in mice administered with DHVD3 [60]. VEGFA is a dimeric glycoprotein that plays a significant role in vascular endothelial cells, primarily through its interactions with the receptors VEGFR1 and VEGFR2 on the endothelial cell membrane [61]. It is considered a mitogen since, through these two receptors (more for VEGFR2 than for VEGFR1), it stimulates the growth of new blood vessels from pre-existing vessels through the formation of tubular structures [62], even in the eye [63]. It is also noteworthy that VEGFR2 shedding has been recently reported as being mediated by TACE and promoted by VEGFA [21]. Therefore, vitamin D3 supplementation may also be considered a new tool to modulate corneal VEGFA signaling.

In conclusion, the data reported here suggest a protective role exerted by vitamin D3 in preventing the development of corneal damage induced by SS. Indeed, vitamin D3 pretreatment ameliorated corneal epithelium morphology and specifically, inhibited corneal TACE levels and activity. As a consequence, the markers of corneal inflammation, derangement, fibrosis and neovascularization were reduced. Overall, these results reinforce the role that this vitamin has in the integrity of tissues and shed light on the possibility that ocular TACE may be a new target of vitamin D3 for concerns relating to SS-associated ocular damage. This further expands the longstanding pharmaceutical industrial research that has attempted to design specific TACE inhibitors to treat inflammatory diseases [64,65].

The limitations of this study that need to be further addressed are those concerning the serum level of 1,25(OH)2D as a reflection of the doses of vitamin D3 taken, which should not be used as the only marker [66] due to the very short half-period (several hours) and the multifactorial regulation of serum concentration levels. Similarly, the possible differences in the responses to supplementation in the form of changes in the levels of the stable and mostly used biomarkers 25(OH) and 1,25(OH)2D should not be excluded, as was described by Seldeen et al. [53]. Due to the labile level of calcitriol in the serum and the use of the 25(OH)D level as a reference point for supplementation in individual groups, there is a high probability that the statistical analysis may look completely different in this paper. Moreover, also vitamin D3 differential absorption and metabolism should be considered as a limitation to the study, with a particular focus on the gender-specific response to vitamin D3. Indeed, since gender is an important discriminant for ocular pathologies [37] and a different response to vitamin D supplementation related to cardiometabolic markers has been reported between males and females [67], a possible differential effect of vitamin D3 pretreatment in SS-corneal damage could be observed in male gender. Further studies are necessary to deepen this hypothesis. Finally, the cellular infiltrates (inflammatory or immune) as possible sources of TNF-α in the corneas of the TSP-1 KO mice, treated or not treated with vitamin D3, need to be further addressed, although this was not specifically the aim of this study conducted.

## Data Availability

All data relevant to the study are included within the article and its Supplementary Materials.

## Supplementary Materials

The following supporting information can be downloaded at: https://www.mdpi.com/article/10.3390/biomedicines11020616/s1: Table S1: Serum DHVD3 levels (ng/mL) in female Balb-c mice at 6 (6w) and 12 weeks (12w) of age; Figure S1: Uncropped images of representative VEGFA and GAPDH Western blotting membranes.
