# Supplementary material for: Oral Administration of Vitamin D3 Prevents Corneal Damage in a Knock-Out Mouse Model of Sjögren’s Syndrome
Source: Biomedicines. 2023 Feb 18;11(2):616. doi: 10.3390/biomedicines11020616 (PMC9953695; doi:10.3390/biomedicines11020616)
Supplement: Supplementary file 1 [file biomedicines-11-00616-s001.zip › biomedicines-2208359-supplementary.pdf]

biomedicines

Oral Administration of Vitamin D3 Prevents Corneal Damage in a Knock-Out Mouse Model of Sjögren's Syndrome

Maria Consiglia Trotta [1,†], Hildegard Herman [2,†], Cornel Balta [2], Marcel Rosu [2], Alina Ciceu [2], Bianca Mladin [2], Carlo Gesualdo [3], Caterina Claudia Lepre [1], Marina Russo [1], Francesco Petrillo [4], Gorizio Pieretti [3], Francesca Simonelli [3], Settimio Rossi [3,*], Michele D'Amico [1,‡] and Anca Hermenean [2,‡]

1 Department of Experimental Medicine, University of Campania "Luigi Vanvitelli", Via Santa Maria di Costantinopoli 16, 80138 Naples, Italy
2 "Aurel Ardelean" Institute of Life Sciences, Vasile Goldis Western University of Arad, 86 Revolutiei Av., 310414 Arad, Romania
3 Multidisciplinary Department of Medical, Surgical and Dental Sciences, University of Campania "Luigi Vanvitelli", Via Luigi de Crecchio 6, 80138 Naples, Italy
4 PhD Course in Translational Medicine, Department of Experimental Medicine, University of Campania "Luigi Vanvitelli", 80138 Naples, Italy
* Correspondence: settimio.rossi@unicampania.it
† These authors contributed equally to this work.
‡ These authors contributed equally to this work.

SUPPLEMENTARY MATERIALS

Table S1. Serum DHVD3 levels (ng/ml) in female Balb-c mice at 6 (6w) and 12 weeks (12w) of age.

Balb-c	Serum DHVD3 (ng/L)
6w	8.5 ± 0.9
12w	9.2 ± 1.2

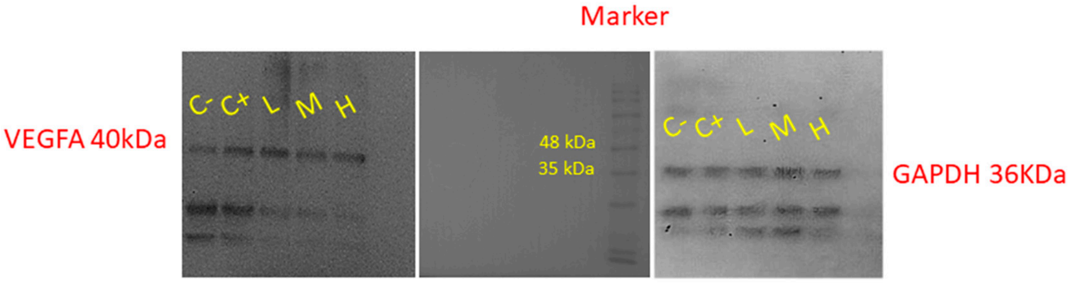

Figure S1. Uncropped images of representative VEGFA and GAPDH Western Blotting membranes.
